# Evaluation of miR-21 and miR-375 as prognostic biomarkers in oesophageal cancer in high-risk areas in China

**DOI:** 10.1007/s10585-016-9828-4

**Published:** 2016-11-24

**Authors:** Yutong He, Jing Jin, LiQun Wang, Yuejiao Hu, Di Liang, Huichai Yang, Yueping Liu, Baoen Shan

**Affiliations:** 1 Cancer Institute, The Fourth Hospital of Hebei Medical University/The Tumor Hospital of Hebei Province, Shijiazhuang, 050011 China; 2 Hospital Medical Insurance Department, The Fourth Hospital of Hebei Medical University/The Tumor Hospital of Hebei Province, Shijiazhuang, China; 3 Pathology Department, The Fourth Hospital of Hebei Medical University/The Tumor Hospital of Hebei Province, Shijiazhuang, China

**Keywords:** Oesophageal cancer, miR-21, miR-375, Prognostic

## Abstract

MicroRNAs have been associated with prognosis in oesophageal cancer (EC), suggesting that miRNAs could play a role in guiding treatment decisions. The aim of this study was to evaluate the prognostic potential of miRNAs found to be associated with zinc deficiency in a geographical area with a high incidence of EC. miRNAs found to be associated with zinc deficiency were isolated from EC cell lines cultured with various Zn levels. The expression levels of the miRNAs were quantified using qRT-PCR. The potential prognostic value of the selected miRNAs was assessed in a cohort study of 88 patients from an area in China with a high incidence of EC. Correlations between miRNAs and patient characteristics were assessed using χ^2^ statistical tests or Fisher’s exact test. A Cox proportional hazards model was used to assess the correlations between miRNAs and overall survival (OS). Forest plots were performed to evaluate the prognostic impact of the miRNAs examined in the present study in the Asian population. The expression levels of miR-21, miR-31, miR-93 and miR-375 were different when Zn levels were varied in EC cell lines, but only miR-21 and miR-375 were associated with patient characteristics and prognosis in patients with EC from an area of China with a high incidence of EC. The patients expressing high levels of miR-21 had poor OS (HR 2.15, 95% CI 1.16–3.97), whereas those with high levels of miR-375 had improved OS (HR 0.47, 95% CI 0.26–0.87).The patients with both a high level of miR-375 and a low level of miR-21 had significantly better outcomes. Forest plots based on an analysis of this Asian population indicated that a high level of miR-21 significantly predicted a shortened OS (HR 1.83, 95% CI 1.42–2.37), whereas a high level of miR-375 was significantly correlated with increased survival (HR 0.56, 95% CI 0.43–0.73). MiR-21 and miR-375 could be used as prognostic biomarkers in areas with a high incidence of EC, and combining these markers may results in a better effect.

## Introduction

Oesophageal cancer (EC) is one of the most common cancers in the world. With an estimated 456,000 new cases and 400,000 deaths per year, EC had the 8th highest incidence of all cancers and the 6th highest cancer-related mortality rate worldwide, based on GLOBOCAN 2012. Approximately 223,000 new cases and 197,200 deaths are associated with EC in China alone.EC therefore accounts for 52.8% of all cases and 49.3% of deaths from oesophageal malignancies worldwide [1]. The incidence of EC varies widely between different regions. For example, it ranges from 3 per 100,000 in low-incidence regions to over 100 per 100,000 in high-incidence areas. Cixian, in Hebei Province, is an area with one of the highest rates of EC in China and the world [2]. Although postoperative recovery and survival rates have substantially improved as a result of continuous progress in diagnostic technologies and therapies, the survival rate remains unsatisfactory, with an approximately 20% overall 5-year survival rate [3]. A multitude of factors influence a prognosis of EC. These include the pathological stage, histological type, pathogenic sites, and biological behaviours of the cancer and the measures used to treat it. However, the influence of some factors has not yet been explored. Because there have been discrepancies in the results of studies examining cancers with the same stage, there is an urgent need for new parameters that can complement differentiation and TNM staging to enable more accurate predictions of prognoses and to provide the best preoperative counselling to patients. To obtain more information regarding the characteristics and survival prospects of patients with ESCC, we focused on the influence of a particular class of microRNAs (miRNAs) on the abnormal expression of genes or proteins [4].

miRNAs are a species of small, conserved endogenous, noncoding, single-stranded RNAs that are approximately 22 nucleotides in length. They act as post-transcriptional regulators by targeting mRNA for degradation or translational repression, usually resulting in gene silencing [5]. They are known to possess the ability to regulate important cellular processes, such as differentiation, the cell cycle, proliferation and apoptosis. By affecting these processes, they can act as tumour suppressors or oncogenes in a variety of cancers. Recent studies have indicated that almost 50% of miRNAs target tumour-associated sensitive sites in genomes, where they can function as either oncogenes or tumour-suppressor genes by recognizing and combining with targeted molecules and either inducing the degradation of the mRNA or post-transcriptionally regulating the translation of the mRNA [6]. A growing amount of evidence suggests that the expression levels of miRNAs in cancer tissues are useful prognostic markers, and changes in the miRNA expression profiles of cancers are being intensively studied in EC. These include miR-16 and miR-21, which are predictors of poor survival in EC [7].

The most common features of regions with a high incidence of EC are an under-developed economy and low nutrient intake. Zinc is an essential trace element, and zinc deficiency (ZD) is a common phenomenon in areas with a high incidence of EC. An inflamed ZD oesophagus has a distinct miRNA signature that resembles the human EC miRNA signature [8]. Louise et al. reported that in individuals with marked ZD, the oesophagus had a 15-microRNA signature in which miR-21, miR-31, and miR-223 were the most up-regulated species [9]. In this study, we examined the levels of a number of miRNAs that are associated with altered Zn levels in EC cell lines and analysed the associations between miRNA levels and survival in 88 patients from an area with a high incidence of EC to explore the possible utility of using ZD-related miRNAs as prognostic biomarkers for EC in high-risk areas.

## Materials and methods

### Cell lines

Human esophageal cancer cell line KYSE170 was obtained from MD Anderson Cancer Center in the United States. The cell line Eca109 was obtained from China Infrastruture of Cell Line Resources. KYSE170 and Eca109 were divided into two groups. One group was cultured in RPMI 1640 medium (Gibco, USA) with 10% foetalbovine serum (FBS, Gibco, USA), and the other was cultured in RPMI 1640 medium with 10% FBS plus ZnSO_4_. We tried 4 levels of ZnSO_4_ (25, 50, 75 and 100 uM/L) for preliminary experiment and chose 50 uM/L in this experiment according to two principles: (1) the level of ZnSO_4_ had no effect on cell status; (2) the level of ZnSO_4_ made most significant changes in miRNA levels. The expression levels of miRNAs were determined for each cell line to analyse the effect that Zn levels had on the miRNAs. The miRNAs that were measured included miR-21, miR-31, miR-93, miR-193a, miR-193b, miR-342 and miR-375, and their relationships with Zn levels were determined using Target Scan Human.

### Patients and samples

This analysis was performed using data obtained from 92 consecutive patients from Cixian, which is a region in Hebei Province with a high rate of histologically confirmed EC. All patients were surgically treated at The Fourth Hospital of Hebei Medical University (also known as The Tumour Hospital of Hebei Province, which is a large and comprehensive level three, grade A hospital) from January 1, 2007 to December 31, 2008. The inclusion criterion was that the patient must have received a pathological diagnosis of primary EC. Surgical samples were obtained within 30 minutes post-operative from each patient and immediately stored in liquid nitrogen. A total of 92 cases that were admitted met the inclusion criterion and only 88 cases with completed follow-up evaluations were included in the study. The follow-up rate was 95.65%. Among these 88 cases, 46 died of EC, whereas 42 were alive on March 15, 2014. The follow-up period ranged from 1 to 88 months, and the median follow-up period was 62 months. Overall, 69 cases were male (78.4%), and 19 cases were female (21.6%). The median age was 67 years old (range 50–87 years old). In addition, the serum zinc levels of patients and healthy controls were measured. This included 10 patients from high and 10 from low incidence areas of EC. The patients were new cases in 2016. The controls were healthy volunteers, including 10 from high and 10 from low incidence areas of EC. This study was approved by the Institutional Human Ethics Committee, and prior informed consent was obtained from all patients. The baseline patient and tumour characteristics are summarized in Table 1.

**Table 1:** Patient and tumor characteristics

Cohort	Cases	%	Deaths	%
Gender				
Male	69	78.4	38	55.1
Female	19	21.6	8	42.1
Age				
<65	31	35.2	19	61.3
≥65	57	64.8	27	47.4
Pathogenic sites				
Upper	5	5.7	2	40.0
Middle	22	25.0	6	27.3
Lower	61	69.3	38	62.3
Pathological type				
ESCC	71	80.7	38	52.8
Other	17	19.3	8	47.1
TNM stage				
I	7	8.0	1	14.3
II	18	20.5	6	33.3
III	63	71.5	39	61.9
Postoperative metastasis				
Yes	38	66.7	22	57.9
No	19	33.3	7	36.8

### Data collection

Personal patient data were extracted from medical records and included gender, age, occupation and family history. Tumour data consisting of the pathogenic site, pathological type, TNM stage and surgical situation were also obtained.

### Follow-up studies

Follow-up evaluations were performed according to the standard follow-up system of the hospital every 6 months after patients were discharged from the hospital. The deadline for follow-up evaluations was March 15, 2014. All patients were followed from the date of histological diagnosis until death or last day of follow-up and were followed for at least 20 months or until death. The survival period was measured from the date of admission to the date of death or to the date of the follow-up deadline.

### Extraction and quantification of miRNA

Total RNA was extracted from tissue samples according to the instructions in the manual form iRNAVanaTM PARISTM (Ambion, TX, USA). miRNA levels were analysed using qRT-PCR with TaqMan, miRNA reverse transcription assays, and the appropriate primers according to the manufacturer’s instructions. Briefly, 10 ng of total RNA was used as the template for 15 µL reverse transcription reactions. Probes were specially designed for specific mature miRNAs. For each miRNA, reactions were performed in triplicate using the 7500 RT-PCR system (Applied Biosystems), and RNU66 (Applied Biosystems, cat. no. 4373382) was used as the normalization control. The sequence used as the forward primer for miR-16(a housekeeping gene) in this study was 5′-CAGCCTAGCAGCACGTAAAT-3′, the sequence used as the reverse primer for miR-16 was 5′-GAGGTATTCGCACCAGAGGA-3′, and the sequence used for the RT reaction was 5′-GTCTCCTCTGGTGCAGGGTCCGAGGTATTCGCACCAGAGGAGACCGCCAA-3′.

The sequence of target gene:

miR-21-3p RT: 5′-CTCAACTGGTGTCGTGGAGTCGGCAATTCAGTTGAGACAGCCCA-3′

miR-21-3p F: 5′-ACACTCCAGCTGGGCAACACCAGTCGATG-3′

miR-31-3p RT: 5′-CTCAACTGGTGTCGTGGAGTCGGCAATTCAGTTGAGATGGCAAT-3′

miR-31-3p F: 5′-ACACTCCAGCTGGGTGCTATGCCAACAT-3′

miR-93-5p RT: 5′-CTCAACTGGTGTCGTGGAGTCGGCAATTCAGTTGAGCTACCTGC-3′

miR-93-5p F: 5′-ACACTCCAGCTGGGCAAAGTGCTGTTCGT-3′

miR-193a RT: 5′-GTCGTATCCAGTGCGTGTCGTGGAGTCGGCAATTGCACTGGATACGACACTGGG-3′

miR-193a F: 5′-GGCCAACTGGCCTACAAAGT-3′

miR-193b RT: 5′-GTCGTATCCAGTGCGTGTCGTGGAGTCGGCAATTGCACTGGATCCGACTCATCT-3′

miR-193b F: 5′-GGCGGGGTTTTGAGGGCG-3′

miR-342 RT: 5′-GTCGTATCCAGTGCGTGTCGTGGAGTCGGCAATTGCACTGGATACGACTCAATC-3′

miR-342 F: 5′-CCGGAGGGGTGCTATCTGT-3′

miR-375-3p RT: 5′-CTCAACTGGTGTCGTGGAGTCGGCAATTCAGTTGAGTCACGCGA-3′

miR-375-3p F: 5′-ACACTCCAGCTGGGTTTGTTCGTTCGGC-3′

All R: TGGTGTCGTGGAGTCG

### Meta-analysis

We searched PubMed, EMBASE, and the Central Registry of Controlled Trials of the Cochrane Library up to April 21, 2016. The following search strategy was employed to both PubMed and EMBASE: microRNA/miR-21, microRNA/miR-375, esophag* cancer/carcinoma/neoplasm. No restrictions on publication type, publication language, or publication year were applied. Cited references from the selected articles were manually searched and assessed. Original research studies that met the following criteria were included: (1) miR-21, miR-375 was determined from human esophageal cancer tissue by quantitative methods; (2) to explore the relationship of miR-21, miR-375 and clinical prognosis of esophageal cancer; (3) There are HR and 95% confidence intervals in the literature or can be extrapolated from the literature. If multiple studies had a duplicate patient cohort, only one study was included for analysis. And the exclusion criteria were: (1) the miR-21, miR-375 and clinical prognosis of esophageal cancer were not studied in human beings; (2) the HR and 95% confidence intervals couldn’t be evaluated from the study; (3) the available outcomes appear in a review.

### Statistical analysis

The SPSS 21.0 and Excel software packages were used for the statistical analysis. We used c2 tests or the Fisher’s exact probability method to determine the relationships between the miRNAs and patient information variables (i.e., gender, age, family history, pathological type, TNM stage, and surgery incidence).OS rates were calculated using the Kaplan–Meier method. The OS rates of the patients in different groups were compared using the log-rank c2 test (inspection level 5, 0.05). Univariate and multivariate Cox regression models were used to determine the factors that influenced OS.

## Results

### Effects of Zn on miRNAs in EC cell lines

**Fig. 1:**
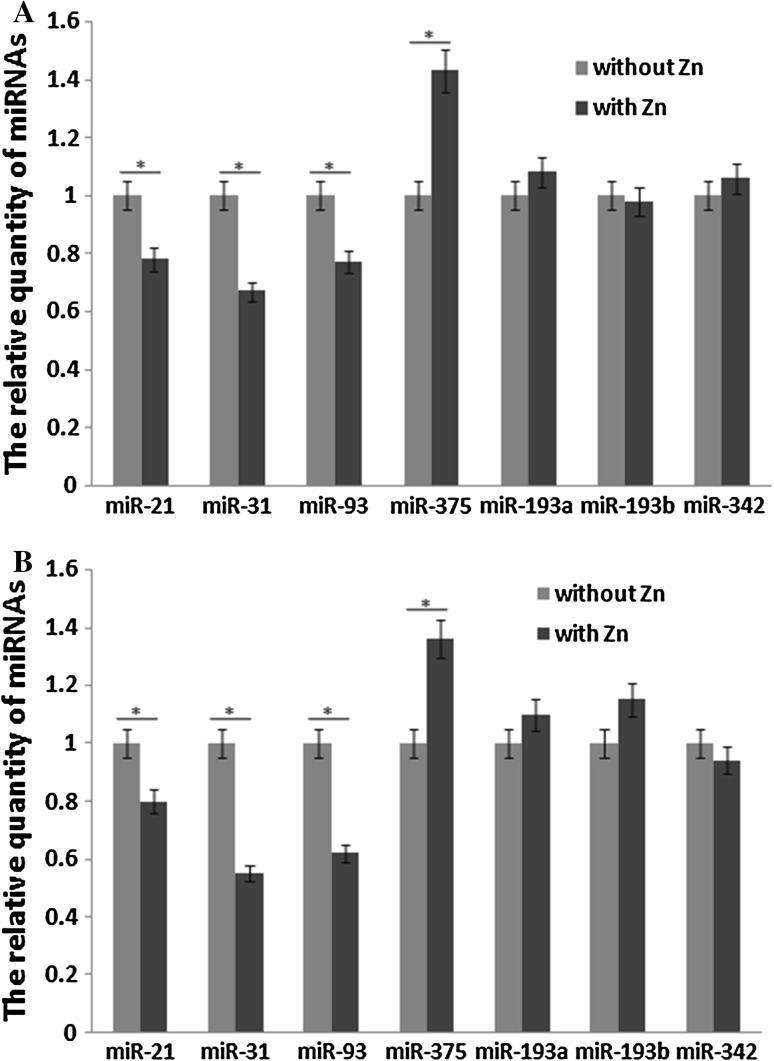
**a** Expression levels of miRNAs in KYSE170 cells grown with and without Zn. The cell line without Zn was cultured in RPMI 1640 medium with 10% foetalbovine serum, and the cell line with Zn was cultured in RPMI 1640 medium with 10% FBS plus 50 µmol/L ZnSO_4_. **b** Expression levels of miRNAs in Eca109 cells grown with and without Zn. The cell line without Zn was cultured in RPMI 1640 medium with 10% foetalbovine serum, and the cell line with Zn was cultured in RPMI 1640 medium with 10% FBS plus 50 µmol/L ZnSO_4_

We compared the expression levels of miRNAs in human EC cell lines that were cultured with or without Zn. We used the KYSE170 and Eca109 cell lines to detect the effects of Zn on the selected miRNAs. The expression levels of the miRNAs in the cells incubated without Zn were used as the standards, and the relative levels of miR-21, miR-31, miR-93 and miR-375 were significantly different (P < 0.05) in the cells incubated with Zn. The expression levels of other miRNAs were not significantly different (Fig. 1). And there was no differences in status between the cells were cultured with or without Zn.

### The zinc levels of patients and control groups in serum

The serum zinc levels of patients and healthy volunteers from high and low incidence areas of EC were measured. The serum zinc level of EC patients from high incidence areas (cancer high group, CH) was 81.00 ± 4.03 ug/100 mL, from low incidence areas (cancer low group, CL) was 83.90 ± 5.51 ug/100 mL; the serum zinc level of control from high incidence areas (normal high group, NH) was 87.20 ± 5.67 ug/100 mL, from low incidence areas (normal low group, NL) was 111.10 ± 5.11 ug/100 mL. We did a LSD analysis and found that the serum zinc level of CH was lower than NH (P = 0.010), of CL was lower than NL (P < 0.001), of NH was lower than NL (P < 0.001) and of CH was no significant differences with CL (P = 0.213).

### Association of the miRNAs with patient characteristics

We sought to determine the relationships between the miRNAs for which expression levels were associated with Zn levels, including miR-21, miR-31, miR-93 and miR-375, with patient characteristics and prognostic values. The expression levels of the miRNAs were determined using a microarray analysis that separated the miRNAs into groups of high and low expressors based on the mean relative cut-off value for each miRNA. The results showed that only miR-21 and miR-375 were associated with patient characteristics and prognoses.

Correlations between miR-21 and patient characteristics were assessed using the χ^2^ statistical test or Fisher’s exact test. The results showed that miR-21 expression was associated with pathogenic sites (P = 0.037): high levels of miR-21 were correlated with upper sites but not with gender (P = 0.229), age (P = 0.722), pathological type (P = 0.191), TNM stage (P = 0.618) and Postoperative metastasis (P = 0.349) (Table 2). The same test was used to analyse the correlations between miR-375 levels and gender (P = 0.506), age (P = 0.704), pathological type (P = 0.708), TNM stage (P = 0.845), Postoperative metastasis (P = 0.190), none of which were correlated with miR-375 expression. However, the pathogenic site was associated with miR-275 in that low miR-375 expression was correlated with upper sites (P = 0.022) (Table 2).

**Table 2:** Association of the miR-21 and miR-375 with patient characteristics

Factor	High miR-21 (n = 42)		Low miR-21 (n = 46)		χ^2^	*P*	High miR-37 (n = 42)		Low miR-375 (n = 46)		χ^2^	*P*
	No.	%	No.	%			No.	%	No.	%		
Gender					1.004	0.229					0.443	0.506
Male	31	44.9	38	55.1			35	50.7	34	49.3		
Female	11	57.9	8	42.1			8	42.1	11	57.9		
Age					0.126	0.722					0.145	0.704
<65	14	45.2	17	54.8			16	51.6	15	48.4		
≥65	28	49.1	29	50.9			27	47.4	30	52.6		
Pathogenic sites					6.610	0.037					7.632	0.022
Upper	4	80.0	1	20.0			1	20.0	4	80.0		
Middle	6	27.3	16	72.7			16	72.7	6	27.3		
Lower	32	52.5	29	47.5			26	42.6	35	57.4		
Pathological type					1.711	0.191					3.366	0.067
ESCC	32	44.4	40	55.6			39	54.2	33	45.8		
Other	10	62.5	6	37.5			4	25.0	12	75.0		
TNM stage					0.964	0.618					0.336	0.845
I	4	57.1	3	42.9			3	42.9	4	57.1		
II	10	55.6	8	44.4			8	44.4	10	55.6		
III	28	44.4	35	55.6			32	50.8	31	49.2		
Postoperative metastasis					0.877	0.349					1.720	0.190
Yes	17	44.7	21	55.3			21	55.3	17	44.7		
No	11	57.9	8	42.1			7	36.8	12	63.2		

### Prognostic value of miR-21 and miR-375 in EC

**Fig. 2:**
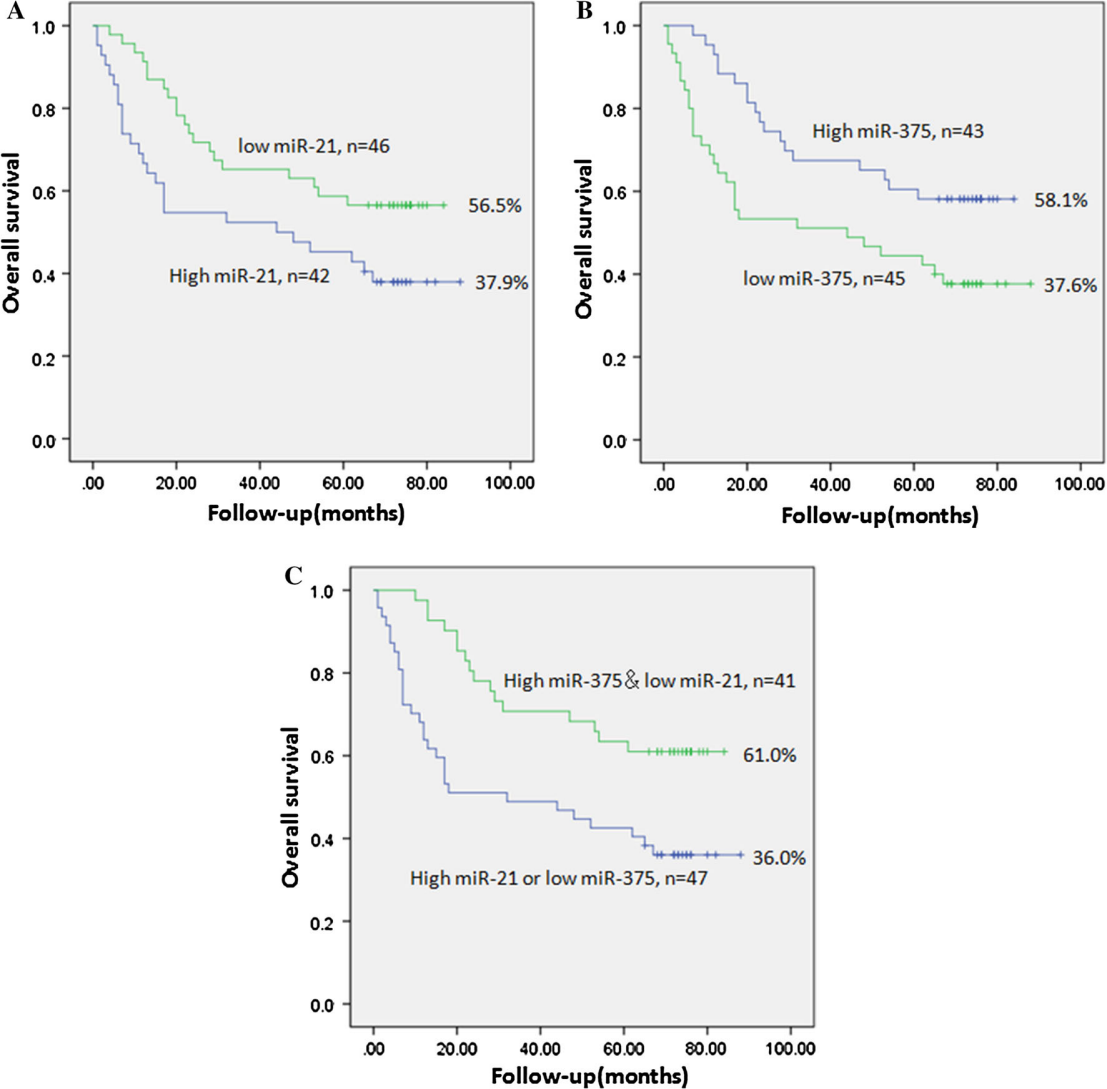
**a** Overall survival in EC patients with high and low miR-21 expression. **b** Overall survival in EC patients with high and low miR-375 expression. **c** Overall survival in EC patients with both high miR-375 expression and low miR-21 expression

A univariate Cox proportional hazards model indicated that both miR-21 (P = 0.032) and miR-375 (P = 0.028) were significantly correlated with OS. The pathogenic site (P = 0.034) and TNM stage (P = 0.013) were also found to be significantly associated with OS (Table 3). The univariate analysis showed that when evaluating OS, better outcomes were observed in EC patients with low miR-21 expression and that patients with high miR-21 expression had worse outcomes (Fig. 2a). Conversely, a trend towards a longer OS was observed in EC patients with high miR-375 expression, and patients with low miR-375 expression had worse outcomes (Fig. 2b).We found that patients with high expression levels of miR-21 were more likely to have low expression levels of miR-375 (Table 4). We then compared survival between (a) patients with high miR-375 expression and low miR-21 expression and patients with either (b) high miR-21 expression or (c) low miR-375 expression. We found that the patients with both high miR-375 expression and low miR-21 expression had significantly better outcomes (Fig. 2c).

**Table 3:** Prognostic factors according to the univariate analysis

Factor	Case	OS rate (%)			*P*
		1 year	3 years	5 years	
Sex					0.323
Male	69	79.7	56.5	44.9	
Female	19	89.5	63.2	57.9	
Age					0.295
<65	31	80.6	58.1	48.4	
≥65	57	82.5	59.6	54.4	
Pathogenic sites					0.034
Upper	5	80.0	60.0	60.0	
Middle	22	90.9	72.7	72.7	
Lower	61	78.7	54.1	44.3	
Pathological type					0.837
ESCC	72	79.2	50.0	47.2	
Other	16	81.3	56.3	50.0	
TNM stage					0.013
I	7	100.0	100.0	85.7	
II	18	94.4	77.8	66.7	
III	63	76.2	49.2	44.4	
Postoperative metastasis					0.143
Yes	38	76.3	57.9	50.0	
No	19	89.5	73.7	63.2	
miR-21					0.039
High	43	69.8	51.2	44.2	
Low	45	93.3	66.7	60.0	
miR-375					0.022
High	43	95.3	67.4	60.5	
Low	45	68.9	51.1	44.4

**Table 4:** Four-fold table for miR-21 and miR-375

MiR-21	MiR-375		Total
	High	Low	
High	2 (2.3%)	40 (45.5%)	42
Low	41 (46.6%)	5 (5.7%)	46
Total	43	45	88

In the univariate analysis, no significant relationships were found between the miRNAs miR-21 and miR-375 and patient characteristics including sex, age, Postoperative metastasis and pathological type. Hence, only pathogenic sites, TNM stage, the expression levels of miR-21 and miR-375 were included in the multivariate analysis. The multivariate analysis indicated that there was a significant association between high expression levels of miR-21 and poor OS in EC patients (HR 2.15, 95% CI 1.16–3.97). A significant correlation was also found between a high level of expression of miR-375 and improved OS in EC patients (HR 0.47, 95% CI 0.26–0.87). In addition, TNM stage was found to be correlated with OS (HR 2.36, 95% CI 1.19–4.70), but no correlation was found between pathogenic sites and survival (HR 1.50, 95% CI 0.78–2.88) (Table 5).

**Table 5:** Prognostic factors according to the multivariate analysis

Factor	*β*	SE	*Wald*	*RR* (95% CI)	*P*
Pathogenic sites	0.405	0.333	1.474	1.499 (0.780–2.880)	0.225
TNM stage	0.859	0.351	5.989	2.361 (1.187–4.696)	0.014
miR-21	0.764	0.314	5.910	2.146 (1.159–3.972)	0.015
miR-375	0.750	0.310	5.842	0.472 (0.257–0.868)	0.016

### Forest plots evaluating the effect of miR-21 and miR-375

**Fig. 3:**
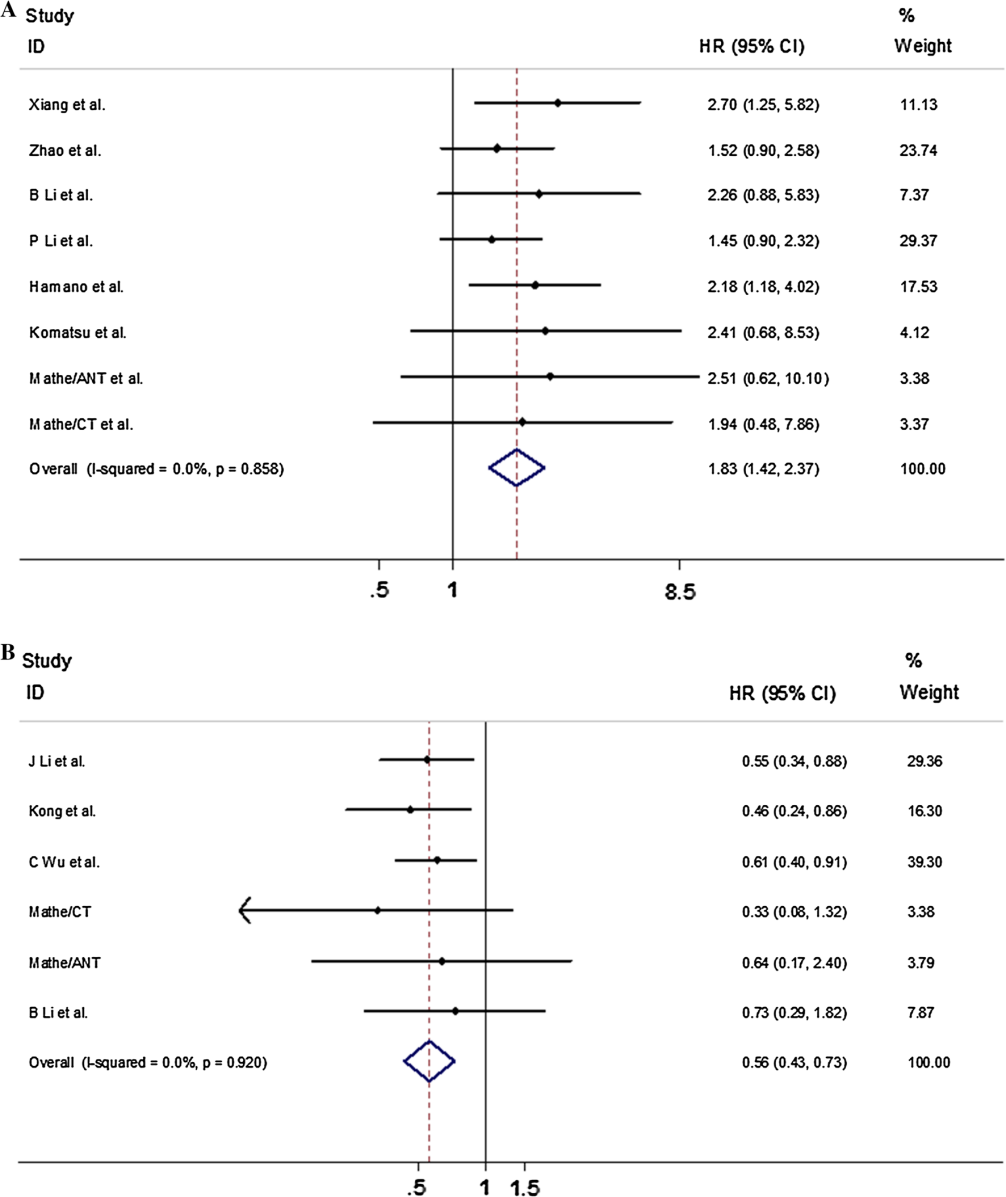
**a** Forest plots evaluating the association between miR-21 expression and prognosis in ES. **b** Forest plots evaluating the association between miR-375 expression and prognosis in ES

The prognostic value of using miR-21 and miR-375 was then evaluated using Forest plot analyses. To investigate the more general prognostic value of miR-21 and miR-375 in Asian populations, we performed Forest plots analyses using data obtained from previously published studies. The characteristics of the included studies were shown in Tables 6 and 7. For miR-21, eight studies were selected for the meta-analysis and 582 patients with esophageal cancer totally. Six studies indicated that there was no significant relationship between the expression level of miR-21 and OS, whereas only two studies reported inverse correlations between miR-21 expression levels and OS. However, although these results were in disagreement, the Forest plot analysis indicated that high expression levels of miR-21 significantly predicted a shortened OS with a pooled HR of 1.83 (95% CI 1.42–2.37) (Fig. 3a). For miR-375, six studies with 318 esophageal cancer patients were selected for inclusion in the Meta-analysis. Of those, three reported that there was no significant correlation between miR-375 expression levels and prognoses, and the other three studies indicated a significant association. The Forest plot analysis resulted in a pooled HR of 0.56 (95% CI 0.43–0.73), indicating that high miR-375 expression was significantly correlated with increased survival (Fig. 3b).

**Table 6:** Characteristics of included studies with miR-21 expression

Study	Year	Population	Sample	Histology	N	Stage	PT	Sample collection	Method	Cutoff	HR	Result	Analysis
Hamano et al.	2011	Japanese	Tissue	ESCC	98	I–IV	Yes	Formalin-fixed	qRT-PCR	Median value	2.18 (1.18–4.02)	OS	NT
Zhao et al.	2012	Chinese	Tissue	ESCC	178	I–III	NG	Frozen	qRT-PCR	3.57-folds	1.52 (0.90–2.58)	OS	multivariate
P Li et al.	2013	Chinese	Tissue	ESCC	76	I–IV	NG	Frozen	qRT-PCR	5-folds	1.45 (0.90–2.32)	DFS	NG
B Li et al.	2015	Chinese	PB	ESCC	38	I–IV	Yes	Frozen	qRT-PCR	Median value	2.26 (0.88–5.83)	OS	NG
Mathe/ANT et al.	2009	MCC	Tissue	SCC, ADC	170	I–IV	Yes	Frozen	qRT-PCR	Median value	2.51 (0.62–10.10)	CH	Univariate and multivariate
Mathe/CT et al.	2009	MCC	Tissue	SCC, ADC	170	I–IV	Yes	Frozen	qRT-PCR	Median value	1.94 (0.48–7.86)	CH	Univariate and multivariate
Komatsu et al.	2012	Japanese	PB	ESCC	50	I–IV	NG	Frozen	qRT-PCR	0.2227 amol/uL	2.41 (0.68–8.53)	OS	multivariate
Xiang et al.	2014	Chinese	Tissue	ESCC	72	I–IV	No	Frozen	qRT-PCR	Median value	2.70 (1.25–5.82)	OS	NG

**Table 7:** Characteristics of included studies with miR-375 expression

Study	Year	Population	Sample	Histology	N	Stage	PT	Sample collection	Method	Cutoff	HR	Result	Analysis
C Wu et al.	2013	Chinese	Tissue	ESCC	20	I–IV	No	Frozen	qRT-PCR	2.71-folds	0.61 (0.40–0.91)	OS	NT
Kong et al.	2011	Japanese	Tissue	ESCC	60	I–IV	NG	Frozen	qRT-PCR	5.38-folds	0.46 (0.24–0.86)	OS	NT
Mathe/ANT et al.	2009	MCC	Tissue	SCC,ADC	170	I–IV	Yes	Frozen	qRT-PCR	Median value	0.64 (0.17–2.40)	CH	Univariate and multivariate
Mathe/CT et al.	2009	MCC	Tissue	SCC,ADC	170	I–IV	Yes	Frozen	qRT-PCR	Median value	0.33 (0.08–1.32)	CH	Univariate and multivariate
B Li et al.	2015	Chinese	Tissue	ESCC	38	I–IV	No	Formalin-fixed	qRT-PCR	Median value	0.73 (0.29–1.82)	OS	NG
J Li et al.	2013	Chinese	Tissue	ESCC	300	I–IV	No	Formalin-fixed	qRT-PCR	Normalized against endogenous 18 s	0.55 (0.34–0.88)	OS	NG

## Discussion

The majority of the worldwide burden of EC occurs in the “Asian Esophageal Cancer Belt,” which extends from northern Iran, east to China and north to Russia. In China, the best-known region for high EC risk is the Taihang Mountain which situated among the Hebei, Henan and Shanxi Provinces. It covered about 90 million people. To our knowledge, this is the first study to assess the potential prognostic value of miRNAs in EC in an area associated with a high-risk of EC. All of the patients in this study came from Cixian in Hebei Province. Cixian and its neighbour Linzhou in Henan Province are the areas with the highest incidence of EC in China and worldwide. The incidence of EC in Cixian is 176.9 per 100,000 in men and 108.8 per 100,000 in women (ASR, W).These rates are much higher than the worldwide incidence (9.0 per 100,000 for men and 3.1 per 100,000 for women) [2].

The major risk factors known to contribute to the development of EC include alcohol consumption, tobacco use and nutritional deficiencies. The most common features of high-risk areas are an under-developed economy, low nutrient intake and limited health resources. Zou et al. reported that zinc intake in high-risk areas was only 72 and 62% of the recommended daily allowance (RDA) in the spring and autumn, respectively, and this may be one of the factors that contribute to the high-risk of EC in these areas [10]. Epidemiological and clinical studies have implicated dietary zinc deficiency in the pathogenesis of EC [11]. Zinc is an essential trace element and a catalytic/structural component that is used by many metalloenzymes and transcription factors. Zinc availability is also important for tumor growth and progression because zinc is a critical component of many enzymes that are involved in hypoxia, angiogenesis, cell proliferation, and cancer metastasis [12]. The Zn content is low in most foods, except for red meat and seafood. People from areas with a high incidence of EC who subsist mainly on a diet that does not include red meat and seafood are likely to be ZD [10]. ZD increases the risk of EC. In a rat model, chronic ZD induced an inflammatory gene signature that fuelled EC development. ZD was found to be a common phenomenon in areas with a high incidence of EC, and an inflamed ZD oesophagus has a distinct miRNA signature that resembles the miRNA profile of human EC, with miR-21 being the most up-regulated species [9]. Changes in miRNA levels may be therefore associated with EC. The ZD-induced modulation of the expression of specific miRNAs has been implicated in the role of ZD in the pathogenesis of diseases [13]. Schetter et al. reported that miR-21 is the most up-regulated oncomiRNA in human cancers and also the most up-regulated species in the ZD oesophagus [14]. ZD induces a pro-oncogenic miRNA signature in the inflammatory oesophagus, and the ZD oesophagus displays a distinct miRNA signature that resembles the miRNA signature of EC [13].

In this study, we first investigated the serum zinc levels of patients and control group from high and low incidence areas of EC. And the results showed that the serum zinc levels of EC patients were lower than healthy people whether in high incidence areas of EC or in low. The serum zinc level of healthy people from high incidence areas of EC was significantly lower than that from low incidence areas could explain the reason of high incidence of EC partly. The expression levels of miRNAs were investigated and miR-21, miR-31, miR-93 and miR-375 were changed when the Zn level was altered in EC cell lines. The expression level of these miRNAs may therefore be subject to alterations that are influence by ZD. The potential prognostic value of these miRNAs was assessed in a cohort study of 88 confirmed EC patients from areas with a high incidence of EC in China. The results showed that only miR-21 and miR-375 were related to prognoses.

There is consensus regarding the notion that miR-21 is an oncogenic miRNA that is up-regulated in a variety of human cancers and that over expression of miR-21 is important in cancer development. MiR-21 is one of the most frequently over expressed miRNAs in cancer, and it exerts its anti-apoptotic effects by targeting the tumor suppressors PDCD4 and PTEN in Certain types of cancer [15–17], but there is no definite conclusion in EC. PDCD4 is one of the most frequently decreased proteins in EC, and it is negatively regulated by miR-21. The down-regulation of PDCD4 has been correlated with tumor stage and nodal metastasis [16]. In addition, some reports have demonstrated that the over expression of miR-21 is correlated with poor clinical outcomes in EC. Recently, a large study evaluated the potential prognostic value of miR-21 in retrospective cohorts of 195 patients in a Western population, and the expression level of miR-21 was significantly correlated with disease-specific survival in patients with EC, while miR-21 was identified as an independent prognostic biomarker [18]. A meta-analysis based on western populations combined four studies, with three of the studies reporting no significant association with survival whereas only one showed a significant and negative correlation between the expression level of miR-21 and survival. A Forest plot analysis confirmed that this relationship had a pooled HR of 1.56 (95% CI 1.18–2.07), indicating that a high expression level of miR-21 significantly predicted a poor prognosis [18].

In our study, the expression level of miR-21 was significantly correlated with OS in patients with EC in both the univariate analysis and the multivariate analysis. The patients with high miR-21 expression levels had worse outcomes, while better OS was observed in the patients with low expression levels of miR-21. MiR-21 was therefore identified as a biomarker of a poor prognosis. In addition, we performed a meta-analysis that was based on an Asian population [7, 15, 18–22].Of the included studies, six found that there was no association between the expression of miR-21 and prognosis, while only two studied found that miR-21 expression levels were significantly associated with survival. However, despite these discordant results, the Forest plot analysis indicated that a high expression level of miR-21 significantly predicted a shortened OS, with a pooled HR of 1.83 (95% CI 1.42–2.37). MiR-21 was indeed found to be an independent marker of a poor prognosis in EC, and this finding was in agreement with our results. The HR for our results, which were obtained by studying an area with a high incidence of EC, was higher than the HR in the Forest plots that were based on an analysis of a Western population or a general Asian population. These results imply that miR-21 may be a better prognostic marker in areas with a high incidence of EC.

MiR-375 is known to play a role as a tumor suppressor, and the expression of miR-375 is reduced in EC as a result of promoter hypermethylation. It has been demonstrated that miR-375 negatively regulates PDK1 and IGF1R and that these effects contribute to the inhibition of tumor proliferation and metastasis [23, 24]. In addition, Mathe et al. reported that a low expression of miR-375 was significantly correlated with a worse prognosis in EAC patients with Barrett’s oesophagus [18]. Inconsistent data have been produced in previous studies regarding the association between the expression of miR-375 and prognoses. In a meta-analysis by Mette that was based on five studies of Western populations, two of the studies reported that there was no association between the expression of miR-375 and prognosis, while three indicated that miR-375 expression levels were significantly associated with survival. A high level of miR-375 was found to be significantly correlated with increased survival in ESCC (HR 0.62; 95% CI 0.49–0.79) [18].

Similarly, miR-375 was associated with patient characteristics and prognoses in this study. Both the univariate analysis and the multivariate analysis demonstrated that there was a trend towards increased survival in patients with high levels of miR-375 (HR 0.47, 95% CI 0.26–0.87). A meta-analysis based on data from studies of Asian populations was performed, and we found that a low level of miR-375 was significantly associated with a worse prognosis (HR 0.56; 95% CI 0.43–0.73) [7, 18, 19, 24–26]. This finding is in disagreement with studies of general Asian populations or Western populations. miR-375 is thought to be a prognostic biomarker that acts as an anti-onco miR. The HR in an area with a high incidence of EC was lower than the HR in the Forest plot analyses, whether they were based on a general Asian population or a Western population. These result simply that miR-375 might be a better prognostic marker in areas with a high incidence of EC.

In addition, we found it interesting that the patients with high levels of miR-21 were more likely to have low expression levels of miR-375. To further verify the prognostic value of miR-21 and miR-375, survival was analyzed in patients with both high miR-375 expression and low miR-21 expression, and the results were compared to survival in patients with either high miR-21 expression or low miR-375 expression. We found that the patients with both high miR-375 expression and low miR-21 expression had significantly better outcomes. These results support the notion that combining analyses of miR-21 and miR-375 levels may have an improved effect in comparison to analyses of either marker alone.

The clinical rational for using prognostic biomarkers, such as miRNAs, is their potential to predict clinical outcomes. This enables clinicians to identify high-risk patients who might benefit from more aggressive treatment strategies and identify low-risk patients who might be safely treated with less intensive treatment regiments. We found that miR-21 and miR-375 are prognostic biomarkers in areas with a high incidence of EC and that they may be used to better effect when combined.
